# Vitamin D-dependent Rickets Type 1A Mimicking Pseudohypoparathyroidism in Presence of Active Tuberculosis

**DOI:** 10.1210/jcemcr/luae176

**Published:** 2024-09-30

**Authors:** Sambit Das, Vishal Agarwal, Binod Prusty, Bijay Ketan Das, Arun Choudhury, Dayanidhi Meher

**Affiliations:** Department of Endocrinology, Kalinga Institute of Medical Sciences, Bhubaneswar 751024, Odisha, India; Department of Endocrinology, Kalinga Institute of Medical Sciences, Bhubaneswar 751024, Odisha, India; Department of Endocrinology, Kalinga Institute of Medical Sciences, Bhubaneswar 751024, Odisha, India; Department of Endocrinology, Kalinga Institute of Medical Sciences, Bhubaneswar 751024, Odisha, India; Department of Endocrinology, Kalinga Institute of Medical Sciences, Bhubaneswar 751024, Odisha, India; Department of Endocrinology, Kalinga Institute of Medical Sciences, Bhubaneswar 751024, Odisha, India

**Keywords:** vitamin D dependent rickets type 1A, pseudovitamin-D-deficiency rickets, *CYP27B1*, rickets, pseudohypoparathyroidism

## Abstract

Vitamin D-dependent rickets type 1A is caused by pathogenic variants of *CYP27B1* gene, which is inherited in autosomal recessive pattern. These variants lead to defective 1α-hydroxylase enzymatic activity, leading to impaired renal formation of 1,25(OH)_2_ vitamin D. We report a case of a 16-year-old Asian male patient, with short stature and progressive bone deformity, whose biochemical parameters revealed low levels of 1,25(OH)_2_ vitamin D, low serum calcium levels, along with high phosphorus and raised levels of intact parathyroid hormone. These biochemical parameters suggested the diagnosis of pseudohypoparathyroidism. The patient also had concurrent extrapulmonary tuberculosis during the time of presentation to our endocrine unit. However, on molecular testing, it was revealed that the patient was harboring pathogenic variants of the *CYP27B1* gene, in a compound heterozygous manner, with a novel missense mutation in exon 6 of the *CYP27B1* gene, c.1136G > C (p.Arg379Thr), suggesting the diagnosis of vitamin D-dependent rickets type 1A. The cause of high phosphorus at the time of presentation, which led to a diagnostic dilemma of pseudohypoparathyroidism, was later explained by presence of active extra pulmonary tuberculosis. This report describes a case of vitamin D-dependent rickets type 1A, mimicking pseudohypoparathyroidism owing to presence of concurrent illness like extrapulmonary tuberculosis.

## Introduction

Rickets and osteomalacia are the prime disorders of bone and mineral metabolism. Prolonged deficiency of minerals like calcium or phosphorus leads to defective mineralization of osteoid [1]. If it occurs in adults, the condition is known as osteomalacia, whereas defective mineralization and development of epiphyseal growth plate occurring in a developing child is known as rickets [2]. Nutritional vitamin D deficiency constitutes the most common etiological cause for rickets worldwide [3]. Other rare causes include defective hydroxylation of vitamin D in hepatocytes and proximal renal tubules leading to defect in vitamin D undergoing 25-α hydroxylation to 25-OH vitamin D and 1-α hydroxylation to form active 1,25(OH)_2_ vitamin D respectively. *CYP2R1* gene located in chromosome 11p15.2 encodes the 25-α hydroxylase enzyme, whereas the *CYP27B1* gene located in chromosome 12q14.1 encodes the 1-α hydroxylase enzyme [4]. Pathogenic variants of the *CYP27B1* gene can lead to 1α-hydroxylase deficiency; the disorder is commonly known as vitamin D-dependent rickets type 1A (VDDR 1A) or pseudo-vitamin D deficiency rickets type 1A [1]. The name “vitamin D dependent rickets” is named so because of the inherit inability of the body to maintain adequate levels of active vitamin D. It is inherited as autosomal recessive disorder. In this report, we describe a case of VDDR 1A, which presented with features of hypocalcemic rickets, but the biochemical parameters resembled that of pseudohypoparathyroidism with high serum phosphate levels because of the presence of concurrent active extrapulmonary tuberculosis.

## Case Presentation

**Figure 1.**
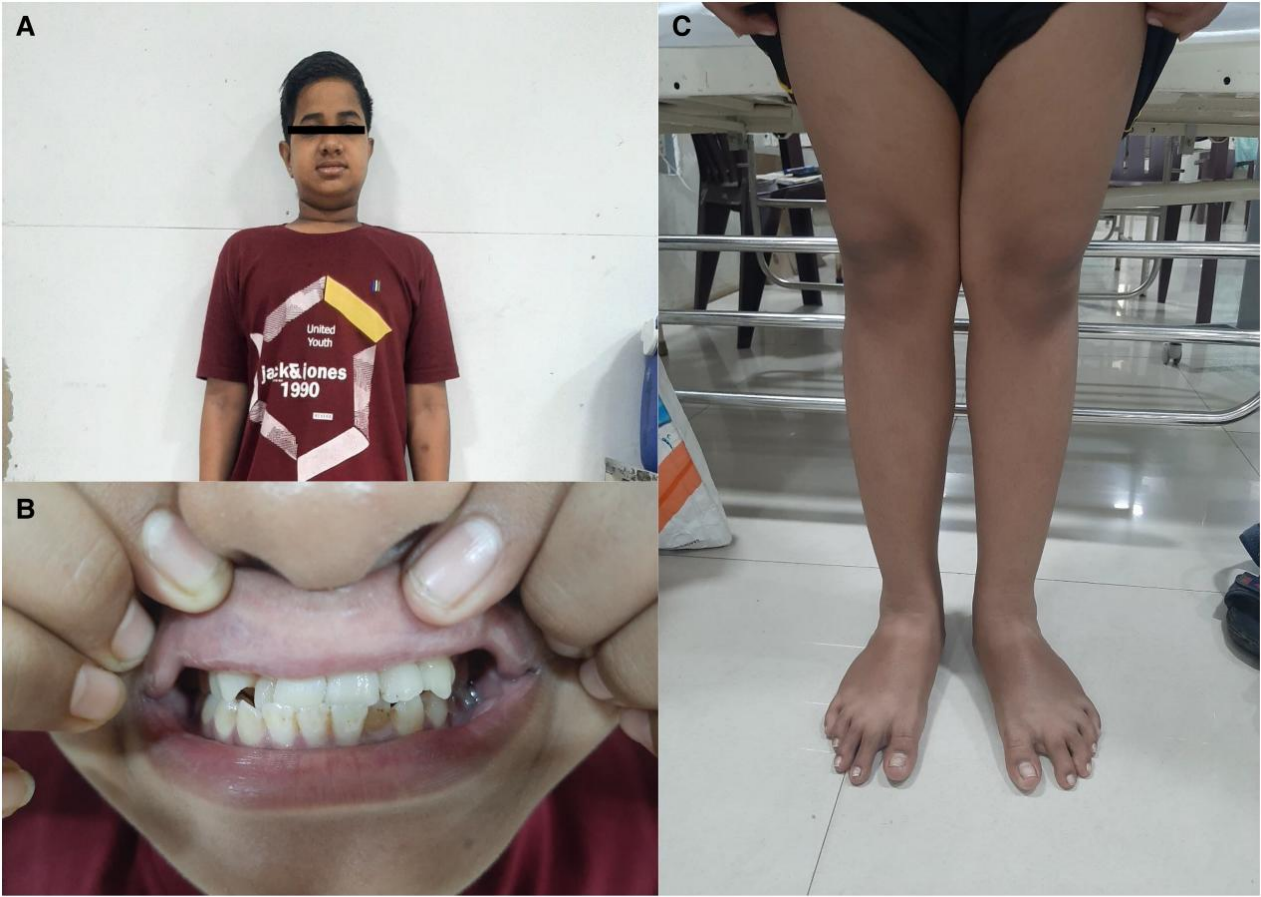
Clinical picture of the patient showing round facies (A), enamel hypoplasia with chalky white appearance of teeth (B), and genu valgum along with sandal toe deformity (C).

We describe a case of a 16-year-old Asian male child, born out of a nonconsanguineous marriage, with normal weight and length at birth. At the age of 1 year, he had a history of delayed motor development milestones and delayed dentition, for which he was referred to a pediatric institution and was prescribed medications in the form of oral vitamin D and calcium supplements. At the age of 7 years, he developed waddling of gait along with progressive bowing of legs and was referred to an endocrine unit. Initial workup showed hypocalcemia, along with hypophosphatemia, with high 25(OH)-vitamin D levels, and elevated intact PTH (iPTH). Initial laboratory data are shown in Table 1. A clinical diagnosis of vitamin D-dependent rickets was initially suspected, and the patient was initiated on oral supplementation of active vitamin D (calcitriol), along with oral calcium tablets started at doses of up to 0.25 μg/day and 1 g/day, respectively. From ages 7 to 15 years, he was lost to follow-up by the pediatric endocrinology unit and the patient had poor compliance to medications during that time. At the age of 16 years, he presented to our institution with features of progressive outward bending of both legs along with carpopedal spasms of 20 days’ duration and fever of 15 days’ duration. At the time of presentation to our institute, the initial laboratory data presented in Table 1 was not made available to the medical team. The patient was not on any form of oral calcium or vitamin D supplementation. Physical examination revealed a height of 141.7 cm (*Z*-score of −3.11), weight of 51.6 kg (*Z*-score of −0.45), and body mass index of 25.7 kg/m^2^. The patient was febrile with an oral temperature of 100.1 °F measured at 4 Pm. He had round facies, enamel hypoplasia, with long bone deformity in the form of genu valgum and sandal toe deformity (Fig. 1). Chvostek and Trousseau signs were positive, indicative of hypocalcemia. The patient also had an enlarged palpable left submandibular group of lymph nodes that were matted and nontender.

**Table 1.** Initial laboratory data (at 7 years of age) of the patient with vitamin D hydroxylation-deficient rickets type 1A

Parameter	Result	Reference range
Serum albumin	4 g/dL (40 g/L)	3.9-4.9 g/dL (39-49 g/L)
Serum phosphorus	**3.5 mg/dL** **(1.1 mmol/L)**	3.7-5.6 mg/dL (1.2-1.8 mmol/L)
Serum calcium	**7 mg/dL** **(1.7 mmol/L)**	8.6-10.3 mg/dL (2.1-2.5 mmol/L)
25-hydroxy vitamin D	**70 ng/mL** **(175 nmol/L)**	20-65 ng/mL (50-162 nmol/L)
PTH (intact)	**247 pg/mL** **(26.2 pmol/L)**	15-65 pg/mL (1.6-6.9 pmol/L)
Serum creatinine	0.7 mg/dL (61 µmol/L)	0.5-1.1 mg/dL (44-97 µmol/L)

Abnormal values are shown in bold font. Values in parentheses are International System of Units (SI).

## Diagnostic Assessment

**Figure 2.**
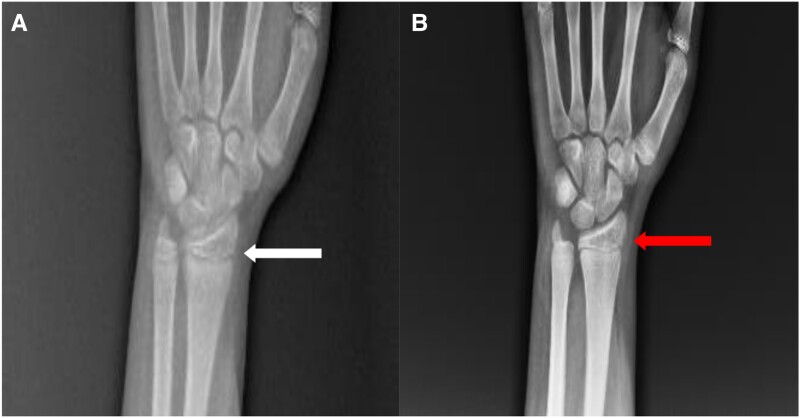
X-rays of left hand wrist joint (anteroposterior view) before (A) and after (B) 6 months of treatment. (A) Metaphyseal fraying and splaying of the distal radial and ulnar metaphyseal (arrow). (B) Resolution of the metaphyseal changes and improvement in deformity after 6 months of therapy (arrow).

Bone radiographs revealed features of active rickets in the form of metaphyseal widening with irregular margins in the epiphyseal end of long bones (Fig. 2A). Laboratory data revealed an elevated iPTH of 19.4 pmol/L (183.5 pg/mL) (reference range, 1.6-6.9 pmol/L; 15-65 pg/mL), low serum calcium of 1.8 mmol/L (7.2 mg/dL) (reference range, 2.2-2.6 mmol/L; 8.6-10.3 mg/dL). Serum phosphate was high with value of 1.6 mmol/L (4.9 mg/dL) (reference range, 0.9-1.5 mmol/L; 2.7-4.7 mg/dL). 25-(OH) D level was normal with value 116.8 nmol/L (46.8 ng/mL) (reference range, 50-162 nmol/L; 20-65 ng/mL), along with low 1,25-(OH)_2_D of 47 pmol/L (19.6 pg/mL) (reference range, 47.8-190.3 pmol/L; 19.9-79.3 pg/mL). The Mantoux test was positive with induration of >10 mm noted at the end of 48 hours. Fine-needle aspiration cytology of lymph nodes revealed features suggestive of tubercular lymphadenitis. Therefore, a clinical diagnosis of pseudohypoparathyroidism along with active extrapulmonary tuberculosis was made. Whole-exome sequencing was sent to confirm the clinical diagnosis, which revealed compound heterozygous pathogenic variants of the *CYP27B1* gene. The first, c.1136G > C (p.Arg379Thr), was a missense variant in exon 6 of the *CYP27B1* gene. It was a novel pathogenic variant of the *CYP27B1* gene. According to American College of Medical Genetics and Genomics criteria, this variant has been classified as a variant of uncertain significance, but the severity of the impact of this variant on the protein is high based on rare exome variant ensemble learner score. The second variant noted was c.1375C > T in exon 8 of the *CYP27B1* gene, which results in the amino acid substitution p.Arg459Cys. This missense variant has been classified as likely pathogenic according to American College of Medical Genetics and Genomics criteria. Therefore, a clinical diagnosis of VDDR 1A along with active extrapulmonary tuberculosis was made.

## Treatment

The patient was started on oral calcitriol and calcium supplementation of 1.25 µg/day and 1.5 g/day, respectively, in 3 divided doses. Antitubercular therapy was initiated in view of active extrapulmonary tuberculosis.

## Outcome and Follow-up

At follow up at 3 months and 6 months after initiation of therapy, the patient's serum calcium and phosphorus levels remained in the normal range along with normalization of iPTH level, as shown in Table 2. Rachitic changes also improved at the end of 6 months of therapy, as shown in Fig. 2B.

**Table 2.** Temporal trends in biochemical investigations of the patient at 16 years of age

Parameter	Day 1 of admission	Day 3 of admission	3 months on therapy	6 months on therapy	Normal value
Hemoglobin	11.8 g/dL (118 g/L)		12.6 g/dL (126 g/L)	12.4 g/dL (124 g/L)	13-15 g/dL (130-150 g/L)
WBC	8100/µL (8.1 × 10^9^/L)		8000/µL (8 × 10^9^/L)		4000-10000/µL (4 × 10^9^-10 × 10^9^/L)
Serum albumin	4 g/dL (40 g/L)		4.2 g/dL (42 g/L)	4.6 g/dL (46 g/L)	3.9-4.9 g/dL (39-49 g/L)
Serum phosphorus	**4.9 mg/dL** **(1.6 mmol/L)**	**5 mg/dL** **(1.6 mmol/L)**	3.9 mg/dL (1.25 mmol/L)	4.6 mg/dL (1.5 mmol/L)	2.7-4.7 mg/dL (0.9-1.5 mmol/L)
Serum calcium	**7.2 mg/dL** **(1.8 mmol/L)**	**7.9 mg/dL** **(1.9 mmol/L)**	8.7 mg/dL (2.2 mmol/L)	9.1 mg/dL (2.3 mmol/L)	8.6-10.3 mg/dL (2.2-2.6 mmol/L)
Serum magnesium	2.2 mg/dL (0.90 mmol/L)		2.0 mg/dL (0.8 mmol/L)	2.2 mg/dL (0.90 mmol/L)	1.6-2.6 mg/dL (0.6-1 mmol/L)
25-hydroxy vitamin D	46.8 ng/mL (117 nmol/L)		44.7 ng/mL (111.7 nmol/L)	44.4 ng/mL (111 nmol/L)	20-65 ng/mL (50-162 nmol/L)
1,25-dihydroxy vitamin D	**19.6 pg/mL** **(47 pmol/L)**				19.9-79.3 pg/mL (47.8-190.3 pmol/L)
PTH (intact)	**183.5 pg/mL** **(19.4 pmol/L)**		**111.1 pg/mL** **(11.7 pmol/L)**	47.5 pg/mL (5 pmol/L)	15-65 pg/mL 1.6-6.9 pmol/L
Serum creatinine	0.5 mg/dL (44 µmol/L)		0.5 mg/dL (44 µmol/L)	0.5 mg/dL (44 µmol/L)	0.5-1.1 mg/dL (44-97 µmol/L)

Abnormal values are shown in bold font. Values in parentheses are International System of Units (SI).

Abbreviation: WBC, white blood cell.

## Discussion

Pseudohypoparathyroidism is a clinical entity arising out from peripheral resistance to PTH [5]. Biochemically, it is characterized by hypocalcemia, hyperphosphatemia, normal vitamin D levels along with high PTH levels. Some patients with pseudohypoparathyroidism can present with rickets, although such presentation is very rare in the reported literature [6]. Such deformities can occur because variable resistance of PTH on the skeletal system. At the time of initial presentation, the current case had features of hypocalcemic rickets in the form of bony deformities and growth retardation. The patient also had recent history of fever for the past 15 days along with left cervical lymphadenopathy. The biochemical profile revealed hypocalcemia, hyperphosphatemia, along with high PTH levels and normal vitamin D levels, resembling pseudohypoparathyroidism. However, on genetic analysis, it was confirmed that the patient was compound heterozygous for *CYP27B1* gene mutations, clinching the diagnosis for VDDR 1A. So, the biochemical parameter that led to the initial diagnostic dilemma of pseudohypoparathyroidism was high serum phosphorus levels. The reason for high serum phosphorus levels in the patient was concurrent presence of extrapulmonary tuberculosis. Tuberculosis has been shown to be associated with various biochemical changes, including serum calcium and phosphorus levels [7]. Many studies have shown that both pulmonary and extrapulmonary tuberculosis can be associated with high serum phosphorus levels [8]. The mechanism that generates high serum phosphorus has been attributed to lipoproteins released from cell membranes via tissue destruction occurring in tuberculosis, that may alter the phosphorus homeostasis; however, the exact mechanism still remains elusive, providing scope for further research and clinical studies [9]. These biochemical derangements usually resolve after the treatment of active tuberculosis, which also occurred in the present case. This case report highlights the importance of interpreting biochemical parameters in clinical practice, in context with patient's comorbid conditions, which may sometimes influence the serum concentration of these biochemical parameters. It also highlights the relationship of vitamin D deficiency and tuberculosis. Studies have shown that vitamin D influences both innate and adaptive immunity. It enhances innate immunity by increasing transactivation of antimicrobial peptides and enhancing autophagy and generation of reactive oxygen species in macrophages. It is also reported to play a role in differentiation of naïve T cells to regulatory T cells, enhance T-helper (Th)2 cell responses, whereas in vitro studies have shown that it suppresses Th1 and Th17 cell responses, indicating its possible role in adaptive immunity [10].

During the months following the initiation of treatment with calcitriol and calcium supplementation along with antitubercular therapy, the patient’s biochemical parameters gradually improved with resolution and hypocalcemia and normalization of serum phosphorus and PTH levels.

The VDDR 1A genotype in our patient was of a compound heterozygous type. There are few cases of vitamin D deficiency rickets misdiagnosed as pseudohypoparathyroidism [11]. However, to our knowledge, this is the first reported case of an inherited vitamin D metabolism that mimicked pseudohypoparathyroidism, in the presence of concurrent illness like tuberculosis.

## Learning Points

- VDDR 1A results from biallelic pathogenic variants in the *CYP27B1* gene, encoding the enzyme 1α-hydroxylase.
- Biochemically, it is characterized by hypocalcemia, hypophosphatemia, and secondary hyperparathyroidism, together with low 1,25-(OH)_2_ vitamin D and normal 25-(OH) vitamin D levels.
- Both pulmonary and extrapulmonary tuberculosis can increase serum phosphorus concentrations. Therefore, serum phosphorus reports must be carefully interpreted in presence of such a comorbid condition.
- Vitamin D deficiency may be associated with increased risk of tuberculosis because it influences both innate and adaptive immune systems.

## Data Availability

Original data generated and analyzed during this study are included in this published article.

## Abbreviations

iPTH: intact PTH
Th: T-helper
VDDR 1A: vitamin D dependent rickets 1A
